# Can we predict the burden of acute malnutrition in crisis-affected countries? Findings from Somalia and South Sudan

**DOI:** 10.1186/s40795-022-00563-2

**Published:** 2022-08-29

**Authors:** Francesco Checchi, Séverine Frison, Abdihamid Warsame, Kiross Tefera Abebe, Jasinta Achen, Eric Alain Ategbo, Mohamed Ag Ayoya, Ismail Kassim, Biram Ndiaye, Mara Nyawo

**Affiliations:** 1 Department of Infectious Disease Epidemiology, Faculty of Epidemiology and Population Health, London School of Hygiene and Tropical Medicine, London, UK; 2 United Nations Children’s Fund, South Sudan Country Office, Juba, South Sudan; 3 United Nations Children’s Fund, Somalia Country Office, Mogadishu, Somalia; 4 East and Southern Africa Regional Office, United Nations Children’s Fund, Nairobi, Kenya

**Keywords:** Malnutrition, Acute malnutrition, Wasting, Undernutrition, South Sudan, Somalia, Food insecurity, Crisis, Humanitarian, Prediction, Statistical model

## Abstract

**Background:**

Sample surveys are the mainstay of surveillance for acute malnutrition in settings affected by crises but are burdensome and have limited geographical coverage due to insecurity and other access issues. As a possible complement to surveys, we explored a statistical approach to predict the prevalent burden of acute malnutrition for small population strata in two crisis-affected countries, Somalia (2014–2018) and South Sudan (2015–2018).

**Methods:**

For each country, we sourced datasets generated by humanitarian actors or other entities on insecurity, displacement, food insecurity, access to services, epidemic occurrence and other factors on the causal pathway to malnutrition. We merged these with datasets of sample household anthropometric surveys done at administrative level 3 (district, county) as part of nutritional surveillance, and, for each of several outcomes including binary and continuous indices based on either weight-for-height or middle-upper-arm circumference, fitted and evaluated the predictive performance of generalised linear models and, as an alternative, machine learning random forests.

**Results:**

We developed models based on 85 ground surveys in Somalia and 175 in South Sudan. Livelihood type, armed conflict intensity, measles incidence, vegetation index and water price were important predictors in Somalia, and livelihood, measles incidence, rainfall and terms of trade (purchasing power) in South Sudan. However, both generalised linear models and random forests had low performance for both binary and continuous anthropometric outcomes.

**Conclusions:**

Predictive models had disappointing performance and are not usable for action. The range of data used and their quality probably limited our analysis. The predictive approach remains theoretically attractive and deserves further evaluation with larger datasets across multiple settings.

**Supplementary Information:**

The online version contains supplementary material available at 10.1186/s40795-022-00563-2.

## Background

In settings affected by crises due to armed conflict, community violence, displacement and/or food insecurity, acute malnutrition is a prominent public health threat that, at the individual level, presents a short-term mortality risk, exacerbates endemic and epidemic infectious diseases and worsens long-term developmental outcomes. Acute malnutrition prevalence among children is also a key summative indicator of crisis severity, as it reflects the wider situation of food security, livelihoods and the public health and social environment [1]. For the purpose of this paper, and in accordance with current Unicef guidance, we refer to acute malnutrition (also commonly known as wasting) as the occurrence of two partially overlapping presentations: *marasmus*, characterised by a recent and severe weight loss, and the rarer but more lethal oedematous form (*kwashiorkor*). Anthropometric indices including weight-for-height or -length, middle-upper arm circumference (MUAC) and presence of bilateral pitting oedema may be combined into continuous indicators (e.g. weight-for-height/length Z-score, relative to the mean of a well-nourished reference population: WHZ) or dichotomised based on thresholds to classify children as severely or moderately acutely malnourished (SAM, MAM), and, at the population level, compute prevalence estimates [2]. Such information helps to assess progress towards national and global targets, identify an appropriate package of food security and nutritional services, estimate resources needed (e.g. treatment caseload), monitor the performance of services and detect changes in crisis severity as part of early warning systems such as the integrated food security phase classification (IPC) [3–5].

Cross-sectional anthropometric surveys among children 6 to 59 months old (mo) are an important component of nutritional surveillance in crisis settings, along with facility-based and programmatic data [6]. Over the past decade, considerable progress has been made to standardise methods and analysis of these surveys. In particular, the Standardised Monitoring and Assessment of Relief and Transitions (SMART) project [7] provides generic study protocols and aides for survey design, training and quality control, as well as the bespoke Emergency Nutrition Software for sample selection, data entry and analysis. SMART surveys, usually implemented at a small geographic scale (e.g. districts or individual camps), are the most common population-based method to measure malnutrition burden in humanitarian response. However, SMART surveys are somewhat burdensome in terms of human and financial resources, require several weeks to plan, implement and report on, and may have limited geographic reach due to insecurity or other access constraints, thereby resulting in potentially biased, untimely, and/or insufficiently granular information. Otherwise put, surveys alone may not adequately support early detection of deteriorating situations and efficient resource allocation [8]. More recently, COVID-19 related restrictions temporarily curtailed SMART survey implementation, just as the pandemic was expected to contribute to a projected doubling in the global population facing food insecurity crisis conditions, and, consequently, a substantial increase in acute malnutrition burden [9].

To complement small-scale nutrition surveys and other surveillance data, and in order to reduce the burden of repeated surveys while also generating timely information on a more regular basis at operationally useful geographical resolution, we explored the performance of predictive statistical models of acute malnutrition burden in Somalia and South Sudan, two crisis-affected countries prominently affected by service access constraints, food insecurity and malnutrition.

## Methods

### Study design

We used a combination of existing datasets collected for programmatic purposes by humanitarian and government actors (see below) to develop and evaluate country-specific models to predict various anthropometric indicators at the resolution of one month and a single administrative level 2 unit (district in Somalia, county in South Sudan), hereafter referred to as a ‘stratum’.

Drawing from an a priori causal framework of factors leading to acute malnutrition (Additional file 1, Figure S5), we identified potential predictor variables collected at the desired resolution and merged these with individual child-level data from SMART surveys designed to be representative of single strata. We fitted various candidate models to a training data subset, and evaluated their predictive accuracy on a validation data subset, as well as on cross-validation.

### Study population and timeframe

For Somalia (including Somaliland and Puntland), we sourced predictor and anthropometric survey data from January 2014 to December 2018 inclusive. During this period, Somalia’s population rose from about 12.8 M to 14.5 M [10]. Surveys were done in 22 (29%) of Somalia’s 75 districts. For South Sudan, the analysis spanned January 2015 to April 2018, and featured surveys from 63 (80%) of the country’s 79 counties, as per 2013 administrative borders. South Sudan’s population declined from 10.2 M to 9.7 M during the period, reflecting refugee movements to neighbouring countries [11].

### Data sources

#### Anthropometric surveys

We accessed reports and raw datasets of 177 SMART surveys from South Sudan (two were excluded due to very unusual values, leaving 175 analysis-eligible), and 167 from Somalia (82 were excluded: 76, mainly done before 2016, were representative of livelihood zones rather than districts, and thus could not be coupled with predictor data; five appeared to have followed a non-representative sampling design; one had no available dataset, leaving 85 analysis-eligible). For each survey, we inspected the report to identify any possible bias sources and, in particular, any reported restriction of the effective sampling frame due to insecurity or inaccessibility (e.g. if a report stated that two out of 12 *boma*, South Sudan’s administrative level 3 unit, could not be included in the sample, we approximated the sampling coverage as 10/12 ≈ 83%). We also rescaled the ENA software-reported quality score for the survey (a composite of several indicators including proportion of outlier values, digit preference and properties of the distribution of observed values, ranging from 0% = best to 50% = worst [12]) to a 0–100% range, where best = 100%. We reanalysed all surveys by converting the raw anthropometric readings (weight, height or length, age, MUAC) into z-score indices as per the World Health Organization 2006 standardised anthropometric distributions using the anthro package in R, flagging and excluding all observations with missing values, <  > 5 z-scores from the mean and/or outside the allowed age range (6-59mo). Lastly, we classified all children into severe acute malnutrition (SAM) or global acute malnutrition (GAM) according to two alternative definitions: (i) bilateral oedema and/or weight-for-height (WHZ) < 3Z (SAM) or < 2Z (GAM); (ii) bilateral oedema and/or MUAC < 115 mm (SAM) or < 125 mm (GAM) [13]. We fitted generalised linear models (binomial for SAM and GAM, gaussian otherwise) with standard errors adjusted for cluster design to verify concordance with point estimates and 95% confidence intervals (CI) contained in the survey reports.

#### Predictors

We developed a causal framework of acute malnutrition (Additional file 1, Figure S5) based on existing evidence and plausibility reasoning. We used this framework to identify factors potentially predicting the outcomes of interest. We searched for candidate predictor data representing these factors online and through contacts with humanitarian actors in both Somalia and South Sudan, the main desirable characteristics of datasets being stratification by stratum and month, and that data be generated routinely for programmatic purposes, i.e. realistically available without further primary data collection. Most datasets had already been sourced as part of similar projects to retrospectively estimate mortality in both countries [10, 11]. Candidate predictors for both Somalia and South Sudan are detailed in Tables 1 and 2, respectively. Each predictor dataset was subjected to data cleaning to remove obvious errors. We excluded predictors that were missing for ≥ 30% of strata or ≥ 30% of months. Remaining completeness problems were resolved through interpolation (humanitarian presence), manual imputation (missing market data points were attributed a weighted average of the geographically nearest market’s value and the mean of all other non-missing markets, with 0.7 and 0.3 weights respectively) and automatic imputation using the mice R package [14] (water price, SAM and MAM treatment quality). To reduce stochastic noise in the time series, we computed three-month window rolling means for all time-varying predictors and applied moderate local spline smoothing to terms of trade or market price variables. Where appropriate, we computed per-population rates using stratum-month population figures previously estimated as part of mortality estimation projects for each country. Briefly, these combine available base estimates (census projections in South Sudan; quality-weighted averages of four alternative sources in Somalia), natural growth assumptions and data on refugee as well as internal displacement to and from each stratum, by month.

**Table 1:** Candidate predictor datasets, Somalia

Predictor	Variable(s)	Domain	Time span of availability	Source(s)	Notes and assumptions
Administrative level	Administrative entity within Somalia	(various)	n/a (static variable)	n/a	Somaliland, Puntland, south-central Somalia
Rainfall	Total rainfall (mm)	Climate	2013 to 2018	Climate Engine (https://clim-engine-development.appspot.com/fewsNet) [15]	
	Mean of Standard Precipitation Index		2016 to 2018		Compares current rainfall with historical averages
Vegetation density	Normalised Difference Vegetation Index	Climate	2013 to 2018	Food Security and Nutrition Analysis Unit—Somalia (FSNAU)	
Incidence of armed conflict events	events per 100,000 population deaths per 100,000 population	Exposure to armed conflict / insecurity	2010 to 2018	Armed Conflict Location & Event Data Project (ACLED, https://www.acleddata.com/) [16]	Meta-data on individual armed conflict events based on extensive review of multi-language media sources and other public information
Incidence of attacks against aid workers	deaths per 100,000 population injuries per 100,000 population	Exposure to armed conflict / insecurity	2010 to 2018	Aid Worker Security Database (AWSD, https://aidworkersecurity.org/incidents)	Data on various types of attacks to aid workers, capturing information from media sources, aid organisations and security actors
Proportion of IDPs	proportion of IDPs among total district population	Forced displacement	2016 to 2018	Estimated by authors as part of a separate mortality study [10]	
Main local livelihood type	Pastoral, agropastoral, riverine and urban	Food security and livelihoods	n/a (static variable)	FSNAU	Assumed to be constant over time
Water price	Price of 200L drum of water in Somali Shillings	Food insecurity and livelihoods	2013 to 2018	FSNAU	
Terms of trade purchasing power index	Kcal equivalent of local cereals that an average local-quality goat can be exchanged for	Food insecurity and livelihoods	2013 to 2018	Calculated by the authors [10] as part of a separate mortality study based on FSNAU price data from 100 sentinel markets	See Annex of citation for more details on calculation.
	Kcal equivalent of local cereals that can be purchased with an average daily labourer wage				
Incidence of admission to nutritional therapeutic services	cases of SAM admitted to treatment services per 100,000 population	Nutritional status	2011 to 2018	Nutrition Cluster, Somalia	Unpublished data
	cases of GAM admitted to treatment services per 100,000 population		2013 to 2018		
Cholera incidence	cases per 100,000 population	Disease burden (epidemic)	2013 to 2018	FSNAU	Suspected and confirmed cases
Measles incidence	cases per 100,000 population	Disease burden (epidemic)	2013 to 2018	FSNAU	Suspected and confirmed cases
Malaria incidence	cases per 100,000 population	Disease burden (endemic)	2013 to 2018	FSNAU	Suspected and confirmed cases
Humanitarian actor presence	Ongoing humanitarian projects per 100,000 population (all sectors)	Humanitarian (public health) service functionality	2010 to 2018	United Nations Office for Coordination of Humanitarian Affairs	Proxy of intensity of humanitarian response Unpublished data
	Ongoing projects per 100,000 population (health, nutrition and water, hygiene and sanitation)				
Food security humanitarian services	Proportion of the population that are a beneficiary of any food security service	Humanitarian (public health) service coverage	Jan 2013 to Apr 2018	Food Security Cluster, Somalia	Unpublished data
	Proportion of the population that are a beneficiary of cash-based food security services	Humanitarian (public health) service coverage			
	Proportion of the population that are a beneficiary of food distributions	Humanitarian (public health) service coverage			
Quality of SAM treatment	Proportion of SAM admissions that exit the treatment programme cured	Humanitarian (public health) service quality	2011 to 2018	Nutrition Cluster, Somalia	Unpublished data

**Table 2:** Candidate predictor datasets, South Sudan

Variable	Value(s)	Domain	Time span of availability	Source(s)	Notes and assumptions
Administrative level	Broad region within South Sudan	(various)	n/a (static variable)	n/a	northeast, northwest, southern
Rainfall	Difference between current rainfall and 10y historical average (mm)	Climate	2014 to 2018	United Nations World Food Programme Food Security Analysis data site ( http://dataviz.vam.wfp.org/seasonal_explorer/rainfall_vegetation/visualizations)	
Incidence of armed conflict events	events per 100,000 population deaths per 100,000 population	Exposure to armed conflict / insecurity	2010 to 2018	Armed Conflict Location & Event Data Project (ACLE, https://www.acleddata.com/) [16]	Meta-data on individual armed conflict events based on extensive review of multi-language media sources and other public information
Incidence of attacks against aid workers	deaths per 100,000 population injuries per 100,000 population	Exposure to armed conflict / insecurity	2010 to 2018	Aid Worker Security Database (AWSD, https://aidworkersecurity.org/incidents)	Data on various types of attacks to aid workers, capturing information from media sources, aid organisations and security actors
Proportion of IDPs	proportion	Forced displacement	2012 to 2018	Estimated by authors as part of a separate mortality study [11]	
Main local livelihood type	agriculturalist, agropastoral, pastoralist, displaced (Protection of Civilians camps only)	Food security and livelihoods	n/a (static variable)	Famine Early Warning Systems Network (FEWS NET) [17]	Assumed to be constant over time
Terms of trade purchasing power index	Kg of white wheat flour that an average medium goat can be exchanged for	Food insecurity and livelihoods	2011 to 2018	CLiMIS portal (http://climis-southsudan.org/)	
Food distributions	metric tonnes per 100,000 population	Food insecurity and livelihoods	2013 to 2018	United Nations World Food Programme	Unpublished data
Incidence of admission to nutritional therapeutic services	cases of SAM admitted to treatment services per 100,000 population	Nutritional status	2015 to 2018	Nutrition Cluster, South Sudan	Unpublished data
	cases of GAM admitted to treatment services per 100,000 population				
Cholera incidence	cases per 100,000 population	Disease burden (epidemic)	2012 to 2018	World Health Organization	Suspected and confirmed cases. No cases reported before 2014. Unpublished data
Measles incidence	cases per 100,000 population	Disease burden (epidemic)	2012 to 2018	World Health Organization	Suspected and confirmed cases. Unpublished data
Humanitarian actor presence	actors per 100,000 population (all sectors; health, nutrition and water, hygiene & sanitation; health only)	Humanitarian (public health) service functionality	2014 to 2018	United Nations Office for Coordination of Humanitarian Affairs	Proxy of intensity of humanitarian response Unpublished data
Acute flaccid paralysis incidence	cases per 100,000 population	Humanitarian (public health) service functionality	2012 to 2018	World Health Organization	Proxy of functionality of public health surveillance
Uptake of measles routine vaccination	doses given per 100,000 population	Humanitarian (public health) service coverage	2012 to 2018	World Health Organization	Assume no value = no routine vaccination taking place
Quality of SAM treatment	Proportion of SAM admissions that exit the treatment programme cured	Humanitarian (public health) service quality	2015 to 2018	Nutrition Cluster, Somalia	Unpublished data
Quality of MAM treatment	Proportion of MAM admissions that exit the treatment programme cured	Humanitarian (public health) service quality	2015 to 2018	Nutrition Cluster, Somalia	Unpublished data

While for both countries data on food security and nutritional therapeutic services were available (Tables 1 and 2) and moderately predictive (data not shown), we ultimately decided to exclude them as candidate predictors for two reasons: (i) we considered that improved prediction could plausibly result in better targeting of these humanitarian services, which in turn would result in improved nutrition, a reverse-causal effect whose future size the model might fail to predict; and (ii) we assumed that end-users would benefit from a model that could be used to predict malnutrition burden even where none of these services were available, e.g. due to access constraints.

### Predictive models

We explored two prediction approaches, as follows.

#### Generalised linear modelling

We first split the data by period into a training set (consisting of approximately the chronologically first 70% of the data) and a ‘holdout’ (i.e. validation) set (the most recent 30%). For each anthropometric indicator, we fitted generalised linear models (GLM) to individual child observations in the training dataset, with robust standard errors to account for the cluster sampling design of most surveys, a quasi-binomial distribution for binary outcomes (SAM, GAM) and a gaussian distribution for continuous outcomes (WHZ, MUAC), which we did not transform as they were normally distributed. We specified model weights as the product of survey quality score and survey sample coverage.

After visual inspection, we categorised continuous predictors, and selected categorical versus continuous versions of these based on linearity of the association and the smallest-possible Chi-square (for binary outcomes) or F-test (continuous outcomes) *p*-value testing whether the univariate model provided better fit than a null model. We also used this *p*-value to select among candidate lags for each predictor; however, we modelled climate variables (rainfall, Normalised Difference Vegetation Index or NDVI) as either the means of the two trimesters, or the mean over the semester prior to each survey observation. We then fitted models consisting of all possible combinations of predictors, and shortlisted the best 10% based on predictive accuracy (lowest mean square error, MSE) of model predictions, relative to observations in the holdout dataset. Predictions were compared with observations by first aggregating all individual-child predictions as yielded by the models to the stratum-month level (as a mean SAM or GAM prevalence, or the mean of continuous anthropometric outcomes, in that stratum-month).

We manually selected the best fixed effects model among these based on relative accuracy on holdout data, accuracy on external data simulated through leave-one-out cross-validation (LOOCV) [18], the plausibility of observed associations, and model parsimony (while the latter characteristic is relatively unimportant for prediction, in practice we wished to avoid users of the model having to collect a large amount of predictor data). Lastly, we explored plausible two-way interactions.

We also fitted mixed models (with stratum as a random effect, given that in both countries surveys were repeated in many districts / counties). The latter, however, offered inconsistent accuracy advantages over fixed effects models on either cross-validation or holdout datasets. Furthermore, we assumed that end users would be most interested in predicting malnutrition prevalence in hard-to-survey districts / counties, i.e. where no a priori random effects would be estimable. For these reasons, we discarded mixed models altogether.

#### Machine learning

After splitting data as above, we used the ranger package [19] to grow random forest (RF) regression models on the training dataset, aggregated at stratum-month level: this approach makes minimal assumptions about data structure; briefly, it partitions the data according to various randomly generated ‘trees’, where each node is defined by a particular value of one of the predictor variables, with branches being the resulting split in the data; the ‘depth’ of each tree is defined by the number of variables that are used to create nodes; randomness is introduced by the choice of variables to build any given tree, values at which splits occur, and the order of variables in the tree structure. The distribution of the outcome arising from the partitions in each tree is compared to the observed data to determine accuracy. RF averages predictions across a large ensemble of trees. We grew RFs with 1000 trees, using all candidate predictors as above, and computed prediction CIs using a jack-knife estimator [20].

### Performance evaluation

For both the GLM and RF approach, we present various metrics of predictive accuracy, for estimation: (i) effective coverage, defined here as the proportion of stratum-months for which the predicted point estimate fell within the 95% or 80%CIs of the observed data; (ii) relative bias, defined as $\frac{1}{n}\sum_{i=1}^{i=n}\frac{{\widehat{y}}_{i}-{y}_{i}}{{y}_{i}}$, where $n$ is the number of stratum-months, ${\widehat{y}}_{i}$ the prediction and ${y}_{i}$ the observation for stratum-month $i$; and (iii) relative precision, namely the mean ratio of predicted stratum-month one-sided 95%CIs to point estimate; and for classification: (iv) sensitivity and (v) specificity of predictions against SAM or GAM prevalence thresholds commonly used in humanitarian response, and adopting observed point estimates as the gold standard. While it is recommended to avoid over-reliance on thresholds and instead examine changes in malnutrition burden over time in light of contextual factors [6], in practice these arbitrary thresholds, introduced about two decades ago [21], are considered when the baseline is unclear to make initial decisions on the most appropriate nutritional and food security interventions package (e.g. management of SAM only versus of SAM *and* MAM; targeted versus ‘blanket’ of generalised food distributions / cash transfers).

For brevity we present only best models for ‘now-casting’ (i.e. prediction of malnutrition based on data collected up to the present). We also explored models for forecasting malnutrition 3 months into the future (i.e. prediction based on data collected up to 3 months previously), but found that these had low performance (data not shown). All analysis was done using R software [22] through the RStudio [23] platform.

## Results

### Anthropometric survey patterns

**Fig. 1:**
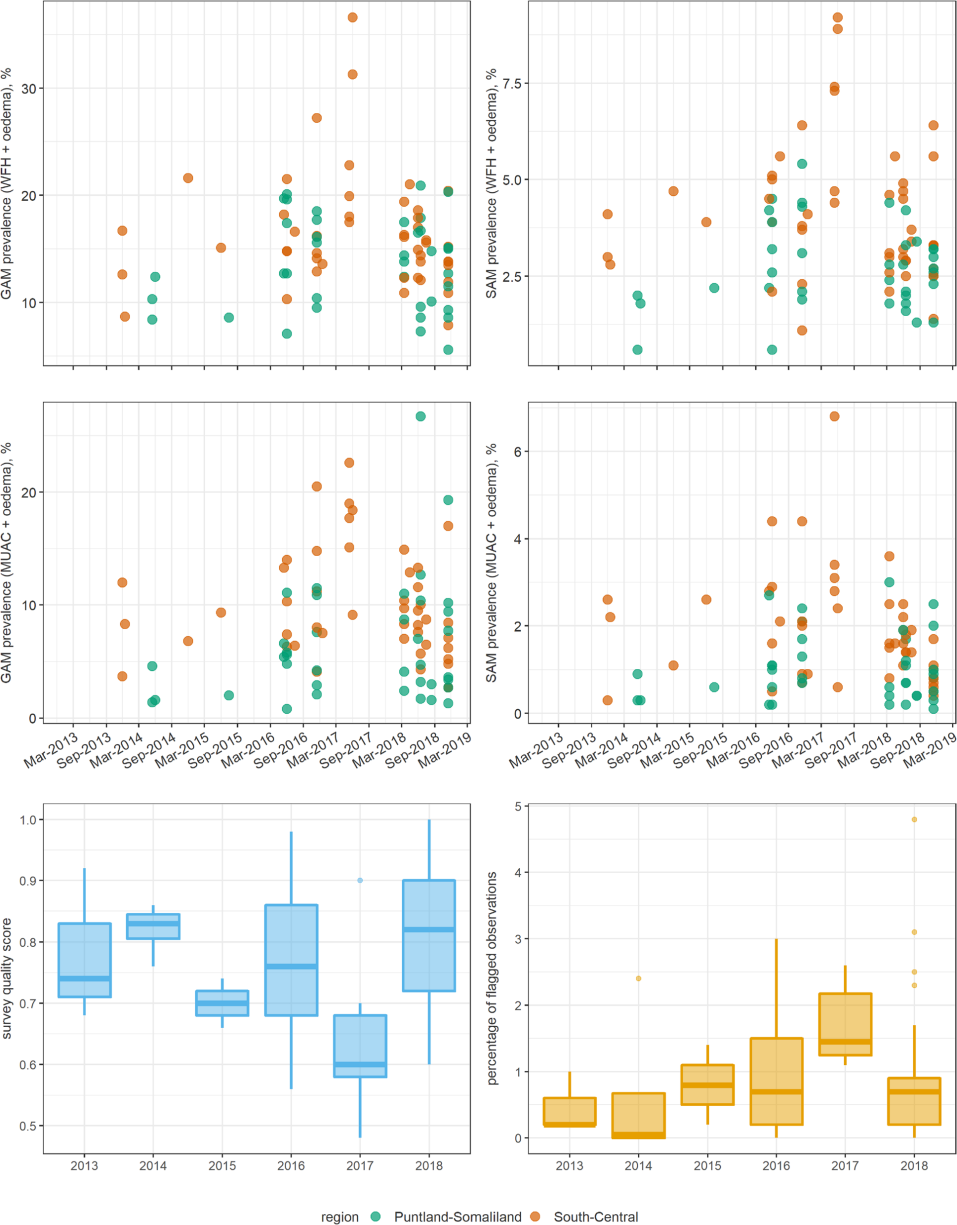
Trends in key survey indicators, Somalia. Each dot represents the point estimate of a single survey. Box plots indicate the median and inter-quartile range, and whiskers the 95% percentile interval

**Fig. 2:**
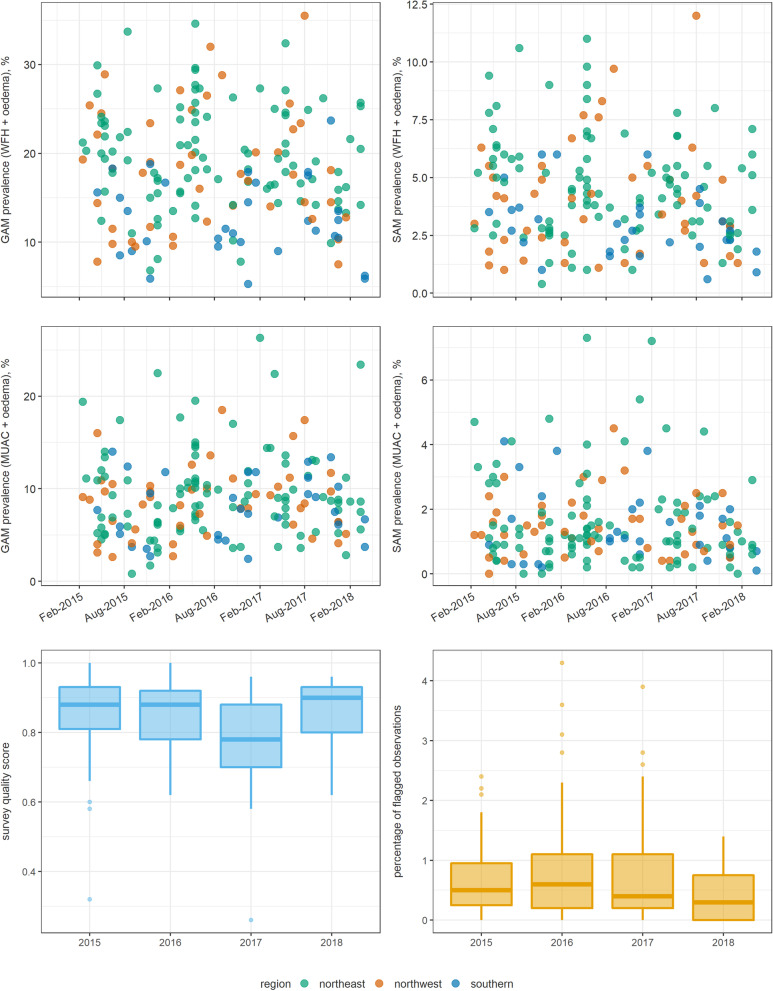
Trends in key survey indicators, South Sudan. Each dot represents the point estimate of a single survey. Box plots indicate the median and inter-quartile range, and whiskers the 95% percentile interval

Details of eligible surveys from Somalia are reported in Table 3 and Fig. 1. Most surveys were done in 2016 and 2018 and the majority relied on multi-stage cluster sampling, with a fairly constant sample size range over time. The highest SAM and GAM prevalence, but also the lowest quality scores, were noted in 2017, during a drought-triggered food insecurity crisis. In South Sudan, all surveys relied on cluster sampling, and there was minimal change in average SAM and GAM prevalence over time; quality scores and the proportion of flagged observations suggested higher survey quality in South Sudan than in Somalia (Table 4, Fig. 2).

**Table 3:** Characteristics of analysis-eligible anthropometric surveys from Somalia. Medians are reported unless noted. Numbers in parentheses indicate the interquartile range

Characteristic	Overall	2013	2014	2015	2016	2017	2018
Eligible surveys (N)	85	3	4	2	25	6	45
Percentage using a cluster sampling design	85.9	100.0	75.0	100.0	80.0	100.0	86.7
Sample size	640 (265 to 1075)	534 (510 to 630)	668 (641 to 833)	683 (501 to 865)	636 (265 to 886)	915 (509 to 1018)	630 (420 to 1075)
GAM prevalence (weight-for-height + oedema), %	14.8 (5.6 to 36.6)	12.6 (8.7 to 16.7)	11.4 (8.4 to 21.6)	11.8 (8.6 to 15.1)	15.6 (7.1 to 27.2)	21.4 (17.5 to 36.6)	14.4 (5.6 to 21)
SAM prevalence (weight-for-height + oedema), %	3.2 (0.6 to 9.2)	3.0 (2.8 to 4.1)	1.9 (0.6 to 4.7)	3.0 (2.2 to 3.9)	3.9 (0.6 to 6.4)	7.3 (4.4 to 9.2)	3.0 (1.3 to 6.4)
GAM prevalence (MUAC + oedema), %	7.6 (0.8 to 26.7)	8.3 (3.7 to 12.0)	3.1 (1.4 to 6.8)	5.7 (2.0 to 9.3)	7.4 (0.8 to 20.5)	18.0 (9.1 to 22.6)	7.6 (1.3 to 26.7)
SAM prevalence (MUAC + oedema), %	1.1 (0.1 to 6.8)	2.2 (0.3 to 2.6)	0.6 (0.3 to 1.1)	1.6 (0.6 to 2.6)	1.3 (0.2 to 4.4)	3.0 (0.6 to 6.8)	1.1 (0.1 to 3.6)
Percentage of flagged observations	0.7 (0.0 to 4.8)	0.2 (0.2 to 1.0)	0.0(0.0 to 2.4)	0.8 (0.2 to 1.4)	0.7 (0.0 to 3)	1.4 (1.1 to 2.6)	0.7 (0.0 to 4.8)

**Table 4:** Characteristics of analysis-eligible anthropometric surveys from South Sudan. Medians are reported unless noted. Numbers in parentheses indicate the interquartile range

Characteristic	Overall	2015	2016	2017	2018
Eligible surveys (N)	175	55	57	52	11
Percentage using a cluster sampling design	100.0	100.0	100.0	100.0	100.0
Sample size	530 (207 to 949)	532 (251 to 790)	523 (325 to 881)	526 (207 to 949)	545 (466 to 768)
GAM prevalence (weight-for-height + oedema), %	17.8 (5.3 to 35.5)	17.8 (5.9 to 33.7)	18.2 (5.3 to 34.6)	17.3 (7.5 to 35.5)	14.2 (5.9 to 25.7)
SAM prevalence (weight-for-height + oedema), %	3.8 (0.4 to 12.0)	4.1 (0.4 to 10.6)	3.9 (1.0 to 11.0)	3.8 (0.6 to 12.0)	3.6 (0.9 to 7.1)
GAM prevalence (MUAC + oedema), %	8.6 (0.8 to 26.3)	6.9 (0.8 to 22.5)	9.3 (2.4 to 19.5)	9.1 (3.6 to 26.3)	7.5 (2.8 to 23.4)
SAM prevalence (MUAC + oedema), %	1.2 (0.0 to 7.3)	1.2 (0.0 to 4.8)	1.2 (0.2 to 7.3)	1.1 (0.2 to 7.2)	0.9 (0.0 to 2.9)
Percentage of flagged observations	0.4 (0.0 to 4.3)	0.5 (0.0 to 2.4)	0.6 (0.0 to 4.3)	0.4 (0.0 to 3.9)	0.3 (0.0 to 1.4)

### Performance of Somalia models

**Fig. 3:**
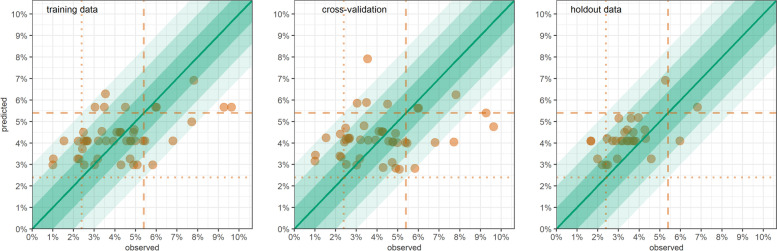
GLM-predicted versus observed SAM (WFH + oedema) prevalence, Somalia, by district-month, on training data, LOOCV and holdout data. Shaded channels indicate an absolute deviance of predictions of up to ±1% (darkest shade), ±2% and ±3% (lightest shade). Vertical dotted lines denote commonly used SAM prevalence thresholds

GLM model coefficients and performance metrics for Somalia are shown in Table 5: odds ratios, OR < 1 and linear coefficients > 0 indicate a protective effect, and vice versa. One predictor (livelihood) consistently featured in the most predictive models (displaced and pastoralist livelihoods were generally associated with better anthropometric status than for agriculturalists). Armed conflict intensity, measles occurrence over the previous trimester, terms of trade, NDVI over the previous semester and average market price of water were useful predictors for some but not all anthropometric outcomes. Generally, predictive performance was low: models yielded mostly upward-biased predictions that fell within the observed survey 95%CIs for only 17% to 80% of stratum-months, depending on the outcome; while denominators were very small, only the model for GAM (WFH + oedema) reached a moderate combination of sensitivity and specificity to classify prevalence as per the 15% threshold. Graphs of predictions versus observations support this pattern; Fig. 3 shows results for SAM (WFH + oedema), while remaining graphs are in the Additional file 1.

**Table 5:** Performance of predictive generalised linear models in Somalia for real-time estimation, by acute malnutrition outcome

Statistic		Categorical outcomes								Continuous outcomes	
		SAM (WFH + oedema)		GAM (WFH + oedema)		SAM (MUAC + oedema)		GAM (MUAC + oedema)		WFH	MUAC
Predictors: coefficient† on training data											
Main local livelihood type											
Agriculturalists		[ref.]		[ref.]		[ref.]		[ref.]		[ref.]	[ref.]
Displaced		0.61*		0.81*		0.44***		0.45***		0.144**	0.337***
Pastoralists		0.80		0.90		0.37***		0.48***		0.105	0.294***
Urban		0.66		0.76		0.62		0.68*		0.121	0.082
Incidence of armed conflict deaths						2-4mths prior		2-4mths prior			2-4mths prior
0						[ref.]		[ref.]			[ref.]
0.1 to 4.9						1.574***		1.81***			-0.291***
≥ 5.0						0.869		0.98			0.057
Terms of trade (cereals : wage)						4-6mths prior					
< 30,000 Kcal						[ref.]					
≥ 30,000 Kcal						0.898					
Measles incidence rate		previous 3mths		previous 3mths							
0		[ref.]		[ref.]							
> 0		1.44***		1.34***							
Mean NDVI		previous 6mths						previous 6mths		previous 6mths	
< 0.20		[ref.]						[ref.]		[ref.]	
≥ 0.20		0.90						0.79**		-0.009	
Mean price of 200L water				3-5mths prior				1-3mths prior		3-5mths prior	1-3mths prior
< 20,000 SOS				[ref.]				[ref.]		[ref.]	[ref.]
≥ 20,000 SOS				1.23***				1.24*		-0.161***	-0.229***
Estimation performance											
Mean square error	training data	0.00028		0.00248		0.00013		0.00177		0.05521	0.10219
	LOOCV	0.00032		0.00345		0.00011		0.00186		0.06923	0.10682
	holdout data	0.00015		0.00206		0.00007		0.00335		0.05765	0.11918
Relative bias	LOOCV	+35.6%		+11.6%		+91.4%		+57.6%		+12.3%	-0.1%
	holdout data	+38.3%		+27.2%		+119.6%		+59.7%		+27.1%	-0.3%
Relative precision of 95%CI	LOOCV	±13.1%		±5.9%		±22.8%		±10.4%		±3.4%	±30.0%
	holdout data	±13.7%		±6.2%		±24.7%		±11.2%		±3.8%	±30.0%
Coverage of 95%CI	LOOCV	58.7%		48.9%		70.2%		45.7%		39.1%	42.6%
	holdout data	80.0%		50.0%		80.0%		30.0%		40.0%	16.7%
Coverage of 80%CI	LOOCV	39.1%		38.3%		44.7%		37.0%		23.9%	25.5%
	holdout data	50.0%		36.7%		43.3%		23.3%		26.7%	16.7%
Classification performance by SAM/GAM prevalence threshold (*n* = denominator of percentage)											
Sensitivity, lower threshold	LOOCV	≥2%	100.0% (40)	≥15%	79.2% (24)	≥2%	50.0% (18)	≥15%	25.0% (4)	n/a	
	holdout data		100.0% (25)		83.3% (12)		50.0% (4)		0.0% (3)		
Sensitivity, upper threshold	LOOCV	≥5%	33.3% (9)	≥20%	12.5% (8)	≥5%	0.0% (1)	≥20%	0.0% (2)		
	holdout data		50.0% (2)		33.3% (3)		n/a (0)		0.0% (1)		
Specificity, lower threshold	LOOCV	<2%	0.0% (6)	<15%	34.8% (23)	<2%	82.8% (29)	<15%	97.6% (42)		
	holdout data		0.0% (5)		27.8% (18)		76.9% (26)		96.3% (27)		
Specificity, upper threshold	LOOCV	<5%	89.2% (37)	<20%	89.7% (39)	<5%	97.8% (46)	<20%	97.7% (44)		
	holdout data		96.4% (28)		96.3% (27)		100.0% (30)		100.0% (29)		

†Odds ratio for categorical outcomes; linear coefficient for continuous outcomes

*0.01 ≤ *p*-value < 0.05 ** 0.001 ≤ *p*-value < 0.01 *** *p*-value < 0.001

RF models had similar performance to the GLM approach. For GAM (WFH + oedema: binary outcome), relative bias, relative precision and 95%CI coverage were +10.1% and + 31.6%, ± 23.0% and ± 17.7%, and 59.6% and 56.7% on LOOCV and holdout data, respectively, with a sensitivity and specificity on LOOCV of 72.0% and 59.1% for the 15% prevalence threshold. The most important variables for prediction were measles incidence, NDVI, terms of trade and water price (Additional file 1). For WFH (continuous outcome), relative bias, relative precision and 95%CI coverage were + 7.1% and + 29.5%, ± 19.1% and ± 13.1%, and 57.4% and 30.0% on LOOCV and holdout data, respectively (Additional file 1).

### Performance of South Sudan models

**Fig. 4:**
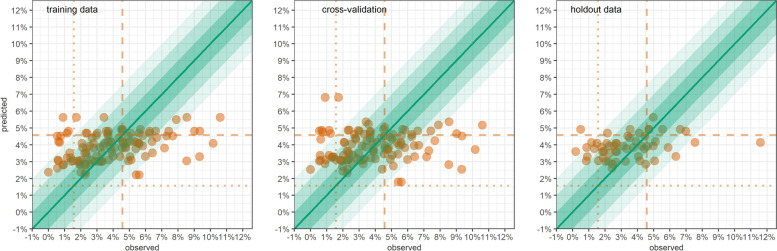
GLM-predicted versus observed SAM (WFH + oedema) prevalence, South Sudan, by district-month, on training data, LOOCV and holdout data. Shaded channels indicate an absolute deviance of predictions of up to ±1% (darkest shade), ±2% and ±3% (lightest shade). Vertical dotted lines denote commonly used SAM prevalence thresholds

Table 6 shows GLM predictions for South Sudan. Here, the most significant associations were with livelihood type, total rainfall and terms of trade. Predictive performance was also low (Fig. 4), with coverage no better than 82% across all outcomes and no instance of high sensitivity and specificity for classification.

**Table 6:** Performance of predictive generalised linear models in South Sudan, by acute malnutrition outcome

Statistic		Categorical outcomes								Continuous outcomes	
		SAM (WFH + oedema)		GAM (WFH + oedema)		SAM (MUAC + oedema)		GAM (MUAC + oedema)		WFH	MUAC
Predictors: coefficient† on training data											
Incidence of acute flaccid paralysis		previous 3mths				previous 3mths		previous 3mths			previous 3mths
0 per 100,000		[ref.]				[ref.]		[ref.]			[ref.]
0.01 to 0.49 per 100,000		0.94				0.80*		0.99			0.08**
≥ 0.50 per 100,000		1.17*				1.19		1.16**			-0.03
Main local livelihood type											
Agriculturalists				[ref.]						[ref.]	[ref.]
Agro-pastoralists				1.46***						-0.30***	-0.21***
Displaced				1.44***						-0.36***	-0.09*
Pastoralists				1.02						-0.15***	-0.13**
Total rainfall		previous 6mths		previous 6mths						previous 6mths	
< 50mm		[ref.]		[ref.]						[ref.]	
50 to 99mm		0.98		0.99						-0.02	
100 to 149mm		0.74***		0.80***						0.11***	
≥ 150mm		0.78		0.97						0.00	
Terms of trade (flour-goat exchange)		3-5mths prior		4-6mths prior						4-6mths prior	
< 20.0Kg		[ref.]		[ref.]						[ref.]	
20.0 to 29.9Kg		0.88*		0.80***						0.15***	
30.0 to 39.9Kg		0.70***		0.84***						0.10***	
≥ 40.0 Kg		0.81**		1.00						0.02	
Incidence of measles				2-4mths prior				1-3mths prior		1-3mths prior	1-3mths prior
0				[ref.]				[ref.]		[ref.]	[ref.]
> 0				1.06				1.16*		-0.04	-0.07*
Doses of measles vaccine administered						3-5mths prior					
0 per 100,000						[ref.]					
0.1 to 99.9 per 100,000						1.24					
100.0 to 199.9 per 100,000						1.21					
200.0 to 299.9 per 100,000						1.74***					
≥ 300.0 per 100,000						1.14					
Estimation performance											
Mean square error	training data	0.00046		0.00297		0.00017		0.00198		0.05358	0.10680
	LOOCV	0.00056		0.00368		0.00020		0.00221		0.06670	0.12667
	holdout data	0.00039		0.00342		0.00009		0.00150		0.06680	0.09943
Relative bias	LOOCV	+41.1%		+12.7%		+80.9%		+38.9%		+9.9%	0.0%
	holdout data	+49.1%		+14.4%		+88.4%		+20.5%		+16.0%	-0.1%
Relative precision of 95%CI	LOOCV	±11.4%		±5.4%		±16.4%		±4.8%		±2.9%	±0.2%
	holdout data	±11.4%		±6.1%		±16.1%		±4.6%		±3.3%	±0.2%
Coverage of 95%CI	LOOCV	64.3%		45.1%		68.7%		49.6%		38.9%	30.4%
	holdout data	66.1%		58.9%		82.1%		57.1%		30.4%	48.2%
Coverage of 80%CI	LOOCV	44.3%		29.2%		47.0%		34.8%		28.3%	26.1%
	holdout data	46.4%		33.9%		60.7%		42.9%		21.4%	33.9%
Classification performance by SAM/GAM prevalence threshold (*n* = denominator of percentage)											
Sensitivity, lower threshold	LOOCV	≥2%	100.0% (97)	≥15%	89.7% (78)	≥2%	28.1% (32)	≥15%	0.0% (9)	n/a	
	holdout data		100.0% (48)		93.8% (32)		27.3% (11)		0.0% (4)		
Sensitivity, upper threshold	LOOCV	≥5%	31.0% (42)	≥20%	60.0% (45)	≥5%	0.0% (3)	≥20%	0.0% (2)		
	holdout data		42.9% (14)		35.3% (17)		n/a (0)		0.0% (2)		
Specificity, lower threshold	LOOCV	<2%	0.0% (18)	<15%	40.0% (35)	<2%	86.7% (83)	<15%	100.0% (106)		
	holdout data		0.0% (8)		25.0% (24)		100.0% (45)		100.0% (52)		
Specificity, upper threshold	LOOCV	<5%	76.7% (73)	<20%	76.5% (68)	<5%	100.0% (112)	<20%	100.0% (113)		
	holdout data		90.5% (42)		87.2% (39)		100.0% (56)		100.0% (54)		

†Odds ratio for categorical outcomes; linear coefficient for continuous outcomes

*0.01 ≤ *p*-value < 0.05 ** 0.001 ≤ *p*-value < 0.01 *** *p*-value < 0.001

RF models had far better fit to the training data than GLMs, but performed similarly on cross-validation and holdout data. The most important variables were livelihood, terms of trade, uptake of measles vaccination and total rainfall (Additional file 1).

## Discussion

In this study we combined a range of previously collected, anthropometric household survey data with a range of potential population-level predictor datasets quantifying theoretical factors causally associated with acute malnutrition burden in crisis settings, to explore whether key quantities such as SAM or GAM prevalence could be estimated through prediction, as a complement to ground surveys. Resulting predictive models based on either GLM or machine learning approaches had disappointing performance in both Somalia and South Sudan across several anthropometric outcomes. Generally, predictive accuracy was better for outcomes based on WFH than on MUAC, but even for the former our models would not, in our opinion, provide actionable information.

Models to predict acute malnutrition risk at the individual or household level exist [24, 25]. While we did not search the literature systematically due to insufficient resources, we are aware of only two other population-level predictive studies. Osgood-Zimmerman et al. [26] produced gridded maps of various anthropometric indicators for all of Sub-Saharan Africa based on periodic countrywide surveys (e.g. Demographic and Health Surveys) and > 20 geospatial remotely sensed or previously estimated predictors; Mude et al. [27] predicted with reasonable accuracy MUAC across time and space in northern Kenya based on village-level data collected for food security surveillance by the Arid Lands Resource Management Project, with predictors including the characteristics of observed MUAC data themselves, cattle herd dynamics, extent of food aid, climate and season. At least one further research project is ongoing (https://www.actionagainsthunger.org/meriam). Bosco et al. [28] have used geospatial and remotely sensed covariates to map stunting prevalence, while Lentz et al. [29] have also demonstrated the potential of a GLM-based approach for predicting food insecurity in Malawi. We have previously used the same datasets as in this study to develop reasonably predictive models of population-level death rate (a farther-downstream and thus potentially even more multifactorial outcome), albeit only for retrospective estimation [10, 11].

Given the above, we expected better predictive performance. It is plausible that additional data on factors causally associated with acute malnutrition, including infant and young child feeding practices, use of food security coping strategies, dietary diversity, access to water, sanitation and hygiene services and health service utilisation would have improved prediction: these data are sometimes generated in crisis settings through cross-sectional surveys, but to our knowledge are not typically available at the granular level required for our predictive problem. It is also likely that problems with available data quality constrained model accuracy. Non-differential error or misclassification arising from measurement problems (e.g. imprecise child anthropometric measurements) and data entry errors would generally reduce model goodness-of-fit and bias estimated associations towards the null: observed-versus-predicted graphs generally suggest ‘regression dilution’ [30], a phenomenon whereby predictions align around an underestimated linear slope, consistent with high noise in predictor variables. Differential error may also have affected model accuracy in various ways. For example, the predictive value of certain variables would have been dampened if anthropometric surveys had systematically underestimated acute malnutrition in the very locations where those predictors exhibited their most extreme values, as might be plausible for surveys done in very remote, insecure locations and thus constrained by time, local staff competency or the need to exclude unreachable communities from the effective sampling frame. We attempted to mitigate such bias by down-weighting lower-quality surveys with evidence of sampling frame selection bias, but models without this weight were not substantively different (data not shown). Pragmatically, these data quality limitations illustrate the challenges of prediction based on data not collected for research.

Our study aim was not to explore associations: as such, we focussed on accuracy and, for example, ignored significant effect modifications that did not improve prediction. Observed GLM associations and variable importance metrics for RF are nonetheless informative. Measles incidence and rainfall or NDVI had plausible associations with most outcomes in both countries, while water price had a very strong association in Somalia. Terms of trade, however, were important in South Sudan but marginal in Somalia. We saw inconsistent associations with forced displacement or armed conflict intensity, though these have been documented elsewhere [31], and, critically, rainfall abnormalities (as opposed to total precipitation) were not an important predictor in any model. A recent review of 90 studies concludes that acute malnutrition is understudied relative to chronic malnutrition (stunting); the review also finds that, while adequate rainfall during the growing season has been associated with less acute malnutrition, relationships with drought and armed conflict are inconclusive [32]. Indeed, the interplay of unusual climate events and armed conflict has proved challenging for food security prediction [33]. More generally, our and others’ findings underscore the context-specific complexity of causal pathways leading to acute malnutrition. They may also reflect the relative noisiness of different datasets, i.e. their accuracy.

Aside from data limitations, our analysis does not thoroughly explore available predictive methods. Among GLM-based approaches, it is possible that different transformations of outcomes or predictors, as well as methods to identify the most informative variables, such as lasso regression, could have yielded improved performance. Among machine learning methods, boosted regression trees could have reduced bias. We note however that these methods would need to yield very considerable improvements over those we used in order to produce useful predictions.

## Conclusions

This analysis suggests that predictive modelling for acute malnutrition burden in crisis settings may not be an immediately viable alternative to ground surveys, at least in the countries studied. Given the potential benefit of such an approach [5], we nonetheless recommend further study, possibly in other settings, using larger datasets and more advanced machine learning methods (boosted regression trees, support vectors, neural networks) and/or Bayesian frameworks. To facilitate such research, as well as other publicly beneficial analyses, humanitarian actors should systematically make key datasets, including but not limited to anthropometric surveys, publicly available in curated, accessible form [34]. These include, but are not limited to, service data from different sectors (e.g. outpatient consultations; vaccination coverage; anthropometric screening data among outpatient children and pregnant women; admissions and exit outcomes for management of acute malnutrition; water availability and quality; coverage of excreta disposal; food security service beneficiaries and Kcal equivalents); market data (e.g. staple prices); morbidity and mortality surveillance data; cross-sectional surveys measuring food security, dietary diversity and infant and young child feeding practices; protection assessments; surveys of perceptions of affected populations; humanitarian presence and activity who-does-what-where matrices; and alternative data on insecurity (e.g. incidents monitored by the UN country team) or humanitarian access (e.g. road safety). A simple principle could be to publish all data barring any whose public availability could place humanitarian actors or affected people at unacceptable risk; aggregation and anonymisation may mitigate such risks. Lastly, any studies to date to predict population-level nutrition burden should be synthesised to identify actionable evidence and guide further analysis.

## Supplementary Information

**Additional file 1:** **Figure S5.** Causal framework for acute malnutrition among children, used to identify potential predictors. **Figure S6.** GLM-predicted versus observed SAM (MUAC + oedema) prevalence, Somalia, by district-month, on training data, LOOCV and holdout data. Shaded channels indicate different absolute deviance of predictions. Vertical dotted lines denote commonly used SAM prevalence thresholds. **Figure S7.** GLM-predicted versus observed GAM (WFH + oedema) prevalence, Somalia, by district-month, on training data, LOOCV and holdout data. Shaded channels indicate different absolute deviance of predictions. Vertical dotted lines denote commonly used GAM prevalence thresholds. **Figure S8.** GLM-predicted versus observed GAM (MUAC + oedema) prevalence, Somalia, by district-month, on training data, LOOCV and holdout data. Shaded channels indicate different absolute deviance of predictions. Vertical dotted lines denote commonly used GAM prevalence thresholds. **Figure S9.** GLM-predicted versus observed mean WFH, Somalia, by district-month, on training data, LOOCV and holdout data. Shaded channels indicate different absolute deviance of predictions. Vertical dotted lines denote potentially useful thresholds. **Figure S10.** GLM-predicted versus observed mean MUAC, Somalia, by district-month, on training data, LOOCV and holdout data. Shaded channels indicate different absolute deviance of predictions. Vertical dotted lines denote potentially useful thresholds. **Table S7.** Performance of random forest models in Somalia, by acute malnutrition outcome. **Figure S11.** RF-predicted versus observed GAM (WFH + oedema) prevalence, Somalia, by district-month, on training data, LOOCV and holdout data. Shaded channels indicate different absolute deviance of predictions. Vertical dotted lines denote commonly used GAM prevalence thresholds. **Figure S12.** RF-predicted versus observed mean WFH, Somalia, by district-month, on training data, LOOCV and holdout data. Shaded channels indicate different absolute deviance of predictions. Vertical dotted lines denote potentially useful thresholds. **Figure S13.** GLM-predicted versus observed SAM (MUAC + oedema) prevalence, South Sudan, by district-month, on training data, LOOCV and holdout data. Shaded channels indicate different absolute deviance of predictions. Vertical dotted lines denote commonly used SAM prevalence thresholds. **Figure S14.** GLM-predicted versus observed GAM (WFH + oedema) prevalence, South Sudan, by district-month, on training data, LOOCV and holdout data. Shaded channels indicate different absolute deviance of predictions. Vertical dotted lines denote commonly used GAM prevalence thresholds. **Figure S15.** GLM-predicted versus observed GAM (MUAC + oedema) prevalence, South Sudan, by district-month, on training data, LOOCV and holdout data. Shaded channels indicate different absolute deviance of predictions. Vertical dotted lines denote commonly used GAM prevalence thresholds. **Figure S16.** GLM-predicted versus observed mean WFH, South Sudan, by district-month, on training data, LOOCV and holdout data. Shaded channels indicate different absolute deviance of predictions. Vertical dotted lines denote potentially useful thresholds. **Figure S17.** GLM-predicted versus observed mean MUAC, South Sudan, by district-month, on training data, LOOCV and holdout data. Shaded channels indicate different absolute deviance of predictions. Vertical dotted lines denote potentially useful thresholds. **Table S8.** Performance of random forest models in South Sudan, by acute malnutrition outcome. **Figure S18.** RF-predicted versus observed GAM (WFH + oedema) prevalence, South Sudan, by district-month, on training data, LOOCV and holdout data. Shaded channels indicate different absolute deviance of predictions. Vertical dotted lines denote commonly used GAM prevalence thresholds. **Figure S19.** RF-predicted versus observed mean WFH, South Sudan, by district-month, on training data, LOOCV and holdout data. Shaded channels indicate different absolute deviance of predictions. Vertical dotted lines denote potentially useful thresholds.

## Data Availability

The data that support the findings of this study are available from various United Nations and non-governmental agencies, but restrictions apply to the availability of these data, which were used under license for the current study, and so are not all publicly available. Data are however available from the authors upon reasonable request and with permission of the above agencies. We have uploaded curated R scripts and all Somalia data on https://github.com/francescochecchi/acute_malnutrition_predictive_models.

## Abbreviations

ACLED: Armed Conflict Location & Event Data Project
AWSD: Aid Worker Security Database
CI: Confidence interval
ENA: Emergency Nutritional Assessment software
FEWS NET: Famine Early Warning Systems Network
FSNAU: Food Security and Nutrition Analysis Unit – Somalia
GAM: Global acute malnutrition
GLM: Generalised linear model
IPC: Integrated Food Security Phase Classification
LOOCV: Leave-one-out cross-validation
MAM: Moderate acute malnutrition
mo: Months old
MUAC: Middle-upper arm circumference
MSE: Mean square error
NDVI: Normalised Difference Vegetation Index
RF: Random forest
SAM: Severe acute malnutrition
SMART: Standardised Monitoring and Assessment of Relief and Transitions
UN: United Nations
WHZ: Weight-for-height Z score
