# Supplementary material for: Can we predict the burden of acute malnutrition in crisis-affected countries? Findings from Somalia and South Sudan
Source: BMC Nutr. 2022 Aug 29;8:92. doi: 10.1186/s40795-022-00563-2 (PMC9421106; doi:10.1186/s40795-022-00563-2)
Supplement: Supplementary file 1 — Additional file 1: Figure S5. Causal framework for acute malnutrition among children, used to identify potential predictors. Figure S6. GLM-predicted versus observed SAM (MUAC + oedema) prevalence, Somalia, by district-month, on training data, LOOCV and holdout data. Shaded channels indicate different absolute deviance of predictions. Vertical dotted lines denote commonly used SAM prevalence thresholds. Figure S7. GLM-predicted versus observed GAM (WFH + oedema) prevalence, Somalia, by district-month, on training data, LOOCV and holdout data. Shaded channels indicate different absolute deviance of predictions. Vertical dotted lines denote commonly used GAM prevalence thresholds. Figure S8. GLM-predicted versus observed GAM (MUAC + oedema) prevalence, Somalia, by district-month, on training data, LOOCV and holdout data. Shaded channels indicate different absolute deviance of predictions. Vertical dotted lines denote commonly used GAM prevalence thresholds. Figure S9. GLM-predicted versus observed mean WFH, Somalia, by district-month, on training data, LOOCV and holdout data. Shaded channels indicate different absolute deviance of predictions. Vertical dotted lines denote potentially useful thresholds. Figure S10. GLM-predicted versus observed mean MUAC, Somalia, by district-month, on training data, LOOCV and holdout data. Shaded channels indicate different absolute deviance of predictions. Vertical dotted lines denote potentially useful thresholds. Table S7. Performance of random forest models in Somalia, by acute malnutrition outcome. Figure S11. RF-predicted versus observed GAM (WFH + oedema) prevalence, Somalia, by district-month, on training data, LOOCV and holdout data. Shaded channels indicate different absolute deviance of predictions. Vertical dotted lines denote commonly used GAM prevalence thresholds. Figure S12. RF-predicted versus observed mean WFH, Somalia, by district-month, on training data, LOOCV and holdout data. Shaded channels indicate different ab [file 40795_2022_563_MOESM1_ESM.docx]

Can we predict the burden of acute malnutrition in crisis-affected countries? Findings from Somalia and South Sudan

ADDITIONAL FILE 1

Francesco Checchi^1^*

Séverine Frison^1^

Abdihamid Warsame^1^

Kiross Tefera Abebe^2^

Jasinta Achen^3^

Eric Alain Ategbo^2^

Mohamed Ag Ayoya^3^

Ismail Kassim^2^

Biram Ndiaye^3^

Mara Nyawo^4^

1 Department of Infectious Disease Epidemiology, Faculty of Epidemiology and Population Health, London School of Hygiene and Tropical Medicine

2 United Nations Children’s Fund, South Sudan Country Office

3 United Nations Children’s Fund, Somalia Country Office

4 United Nations Children’s Fund, East and Southern Africa Regional Office

* Corresponding author: [Francesco.checchi@lshtm.ac.uk](mailto:Francesco.checchi@lshtm.ac.uk)

## Causal framework for acute malnutrition

Figure S5. Causal framework for acute malnutrition among children, used to identify potential predictors.

## Predictive accuracy of additional Somalia models


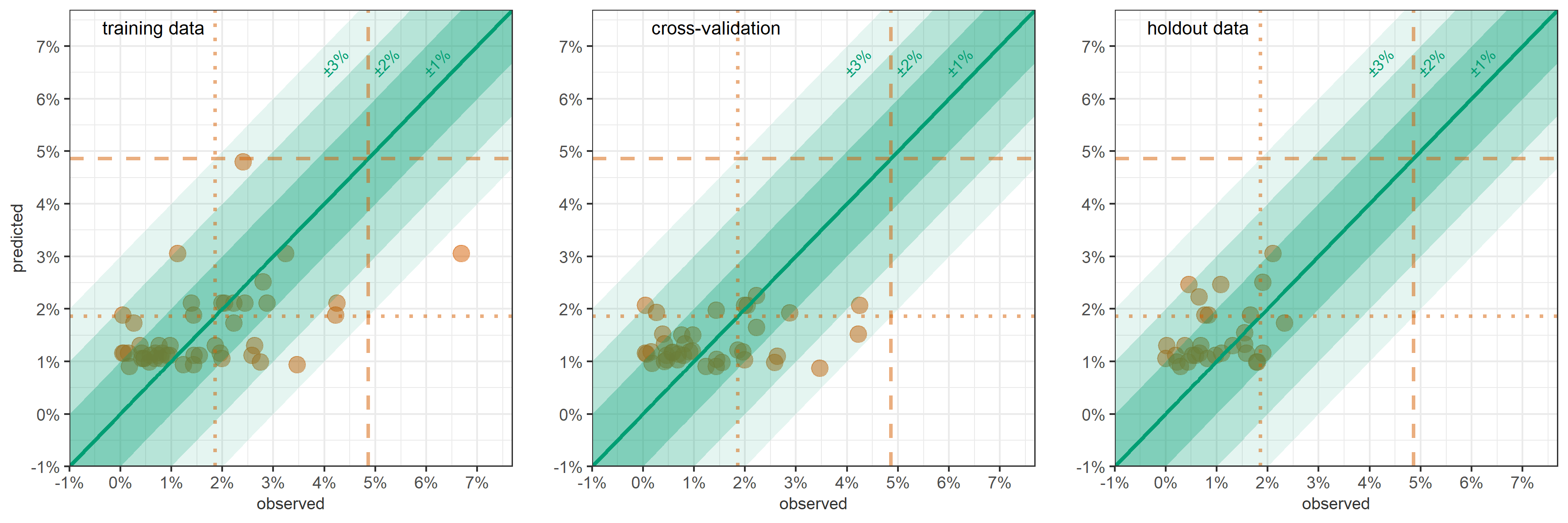


Figure S6. GLM-predicted versus observed SAM (MUAC + oedema) prevalence, Somalia, by district-month, on training data, LOOCV and holdout data. Shaded channels indicate different absolute deviance of predictions. Vertical dotted lines denote commonly used SAM prevalence thresholds.


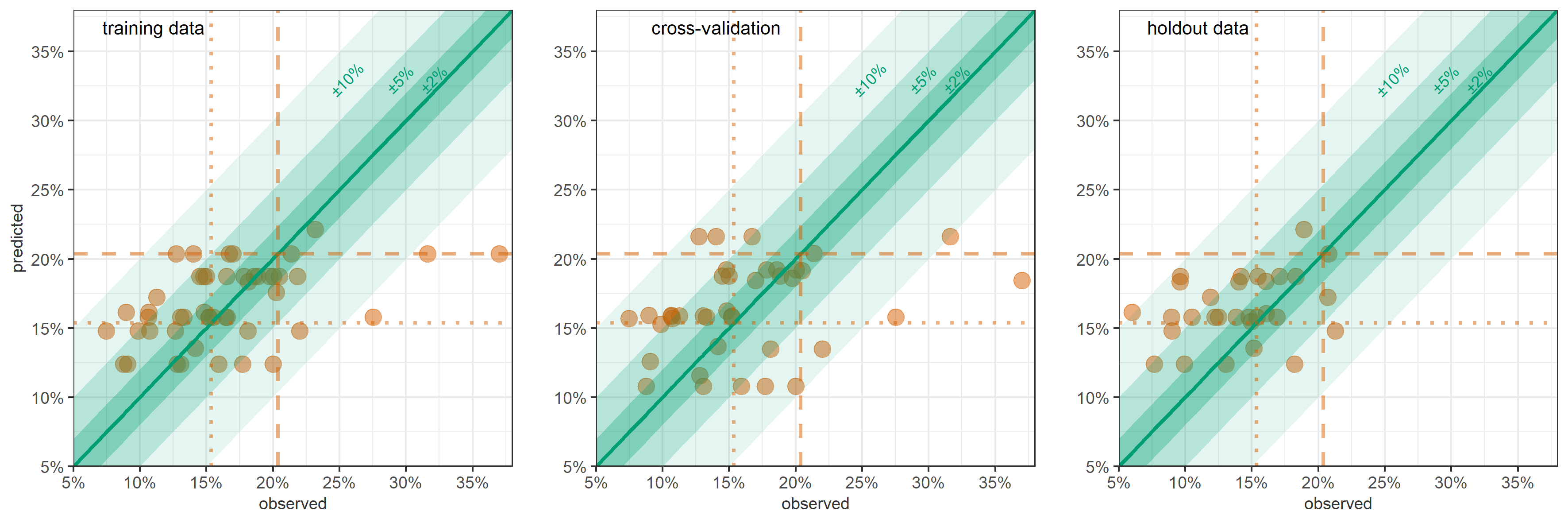


Figure S7. GLM-predicted versus observed GAM (WFH + oedema) prevalence, Somalia, by district-month, on training data, LOOCV and holdout data. Shaded channels indicate different absolute deviance of predictions. Vertical dotted lines denote commonly used GAM prevalence thresholds.


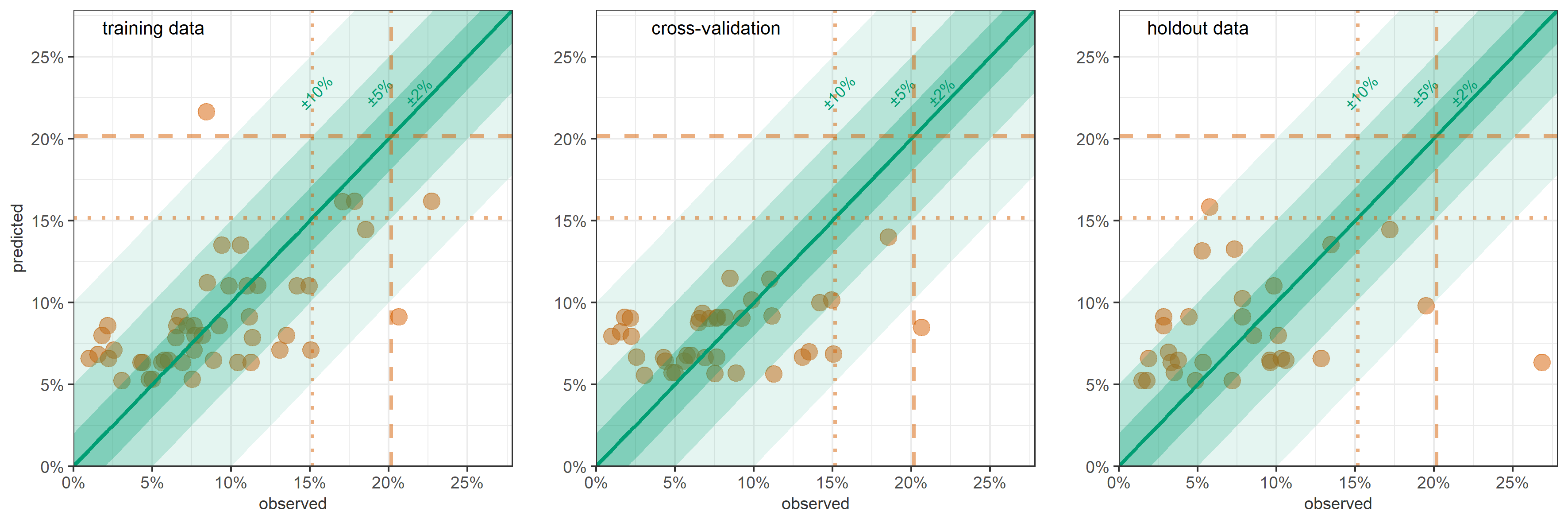


Figure S8. GLM-predicted versus observed GAM (MUAC + oedema) prevalence, Somalia, by district-month, on training data, LOOCV and holdout data. Shaded channels indicate different absolute deviance of predictions. Vertical dotted lines denote commonly used GAM prevalence thresholds.


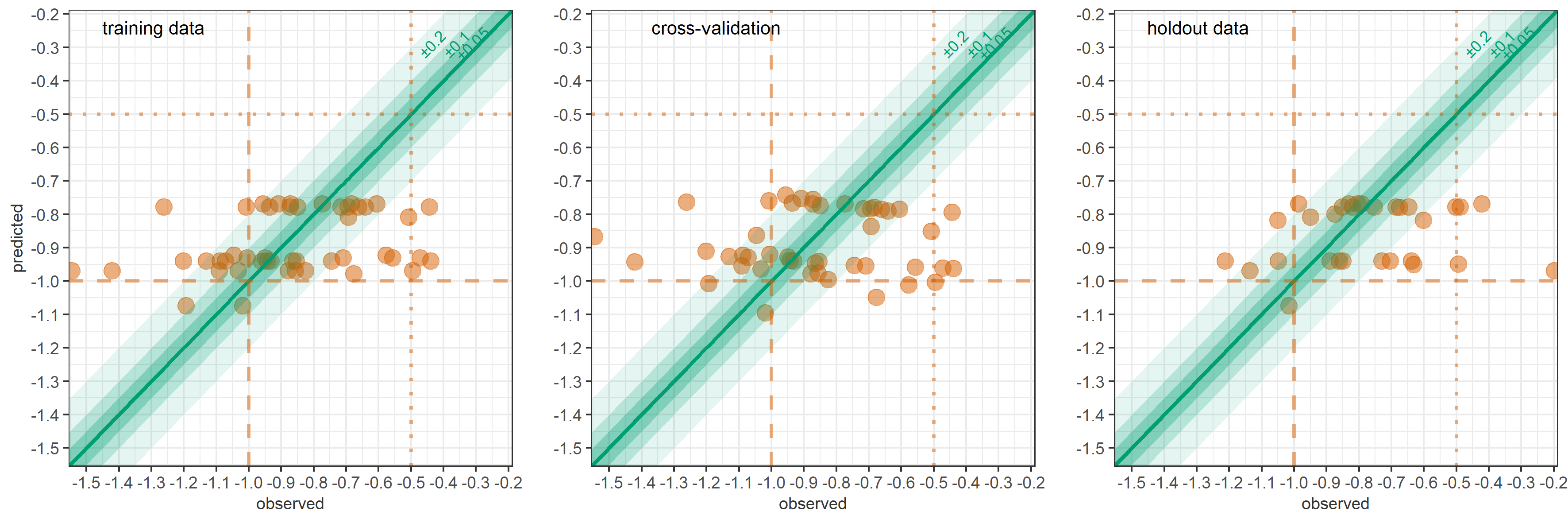


Figure S9. GLM-predicted versus observed mean WFH, Somalia, by district-month, on training data, LOOCV and holdout data. Shaded channels indicate different absolute deviance of predictions. Vertical dotted lines denote potentially useful thresholds.


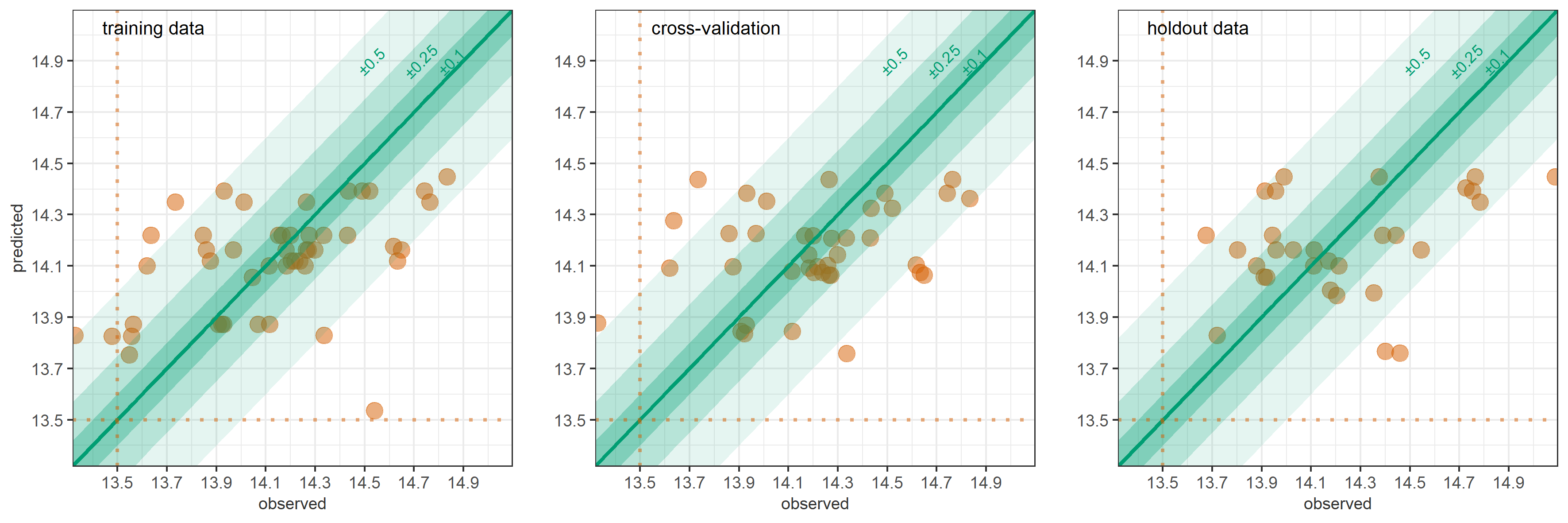


Figure S10. GLM-predicted versus observed mean MUAC, Somalia, by district-month, on training data, LOOCV and holdout data. Shaded channels indicate different absolute deviance of predictions. Vertical dotted lines denote potentially useful thresholds.

Table S7. Performance of random forest models in Somalia, by acute malnutrition outcome.

| Statistic | | Binary:  GAM (WFH + oedema) | | Continuous:  WFH | |
| --- | --- | --- | --- | --- | --- |
| Estimation performance | | | | | |
| Mean square error | training data | 0.00060 | | 0.01016 | |
|  | LOOCV | 0.00299 | | 0.05339 | |
|  | holdout data | 0.00269 | | 0.06746 | |
| Relative bias | LOOCV | +10.1% | | +7.1% | |
|  | holdout data | +31.6% | | +29.5% | |
| Relative precision of 95%CI | LOOCV | ±23.0% | | ±19.1% | |
|  | holdout data | ±17.7% | | ±13.1% | |
| Coverage of 95%CI | LOOCV | 59.6% | | 57.4% | |
|  | holdout data | 56.7% | | 30.0% | |
| Coverage of 80%CI | LOOCV | 44.7% | | 46.8% | |
|  | holdout data | 23.3% | | 26.7% | |
| Classification performance by GAM prevalence threshold (n = denominator of percentage) | | | | | |
| Sensitivity  (lower threshold) | LOOCV | ≥15% | 72.0% (25) | n/a | |
|  | holdout data |  | 66.7% (12) |  |  |
| Sensitivity  (upper threshold) | LOOCV | ≥20% | 0.0% (8) |  |  |
|  | holdout data |  | 33.3% (3) |  |  |
| Specificity  (lower threshold) | LOOCV | <15% | 59.1% (22) |  |  |
|  | holdout data |  | 33.3% (18) |  |  |
| Specificity  (upper threshold) | LOOCV | <20% | 92.3% (39) |  |  |
|  | holdout data |  | 85.2% (27) |  |  |
| Top ten predictors by importance | lag | Importance | p-value | Importance | p-value |
| Measles incidence rate | 1-3mths prior | 0.0003 | 0.050 | 0.0056 | 0.050 |
|  | previous 3mths | 0.0002 | 0.069 | 0.0078 | 0.012 |
| Cholera incidence rate | previous 3mths | not among top ten predictors | | 0.0033 | 0.089 |
| Water price | 3-5mths prior | 0.0001 | 0.434 | 0.0027 | 0.139 |
|  | 2-4mths prior | 0.0002 | 0.356 | 0.0022 | 0.307 |
|  | 1-3mths prior | 0.0002 | 0.327 | 0.0013 | 0.475 |
|  | previous 3mths | <0.0001 | 0.465 | 0.0020 | 0.238 |
| Terms of trade (wage) | 4-6mths prior | 0.0001 | 0.386 | not among top ten predictors | |
|  | 3-5mths prior | 0.0001 | 0.554 | 0.0016 | 0.475 |
|  | 2-4mths prior | <0.0001 | 0.535 | 0.0022 | 0.307 |
| NDVI | previous 6mths | 0.0002 | 0.079 | 0.0029 | 0.089 |


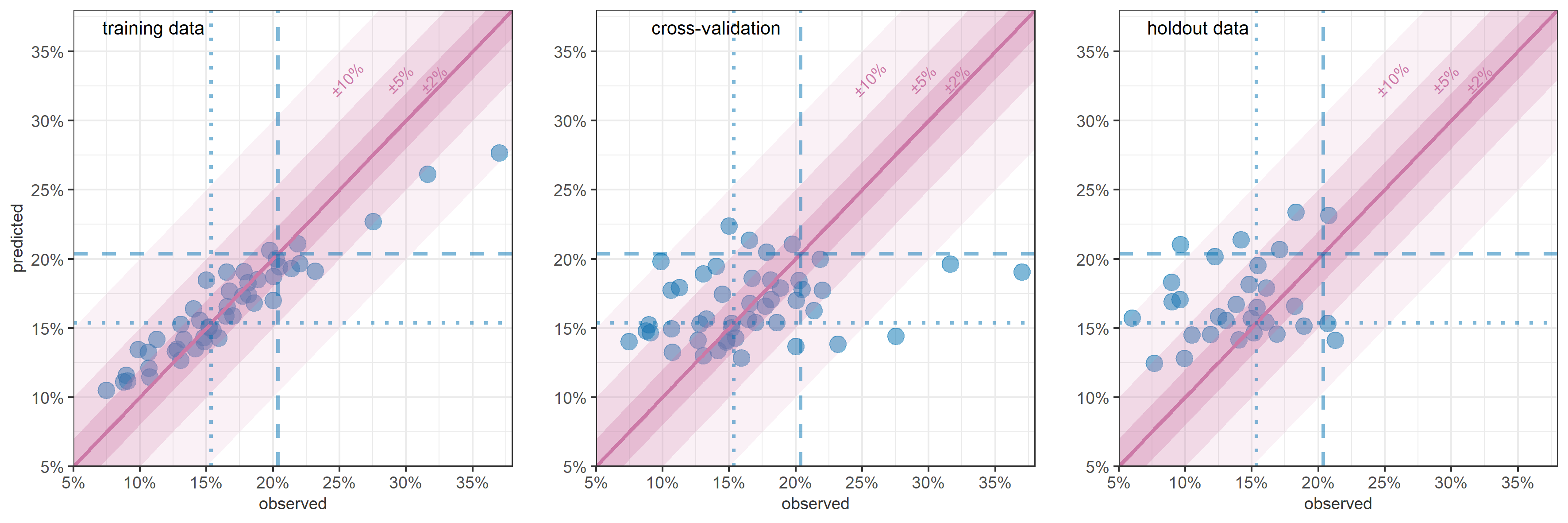


Figure S11. RF-predicted versus observed GAM (WFH + oedema) prevalence, Somalia, by district-month, on training data, LOOCV and holdout data. Shaded channels indicate different absolute deviance of predictions. Vertical dotted lines denote commonly used GAM prevalence thresholds.


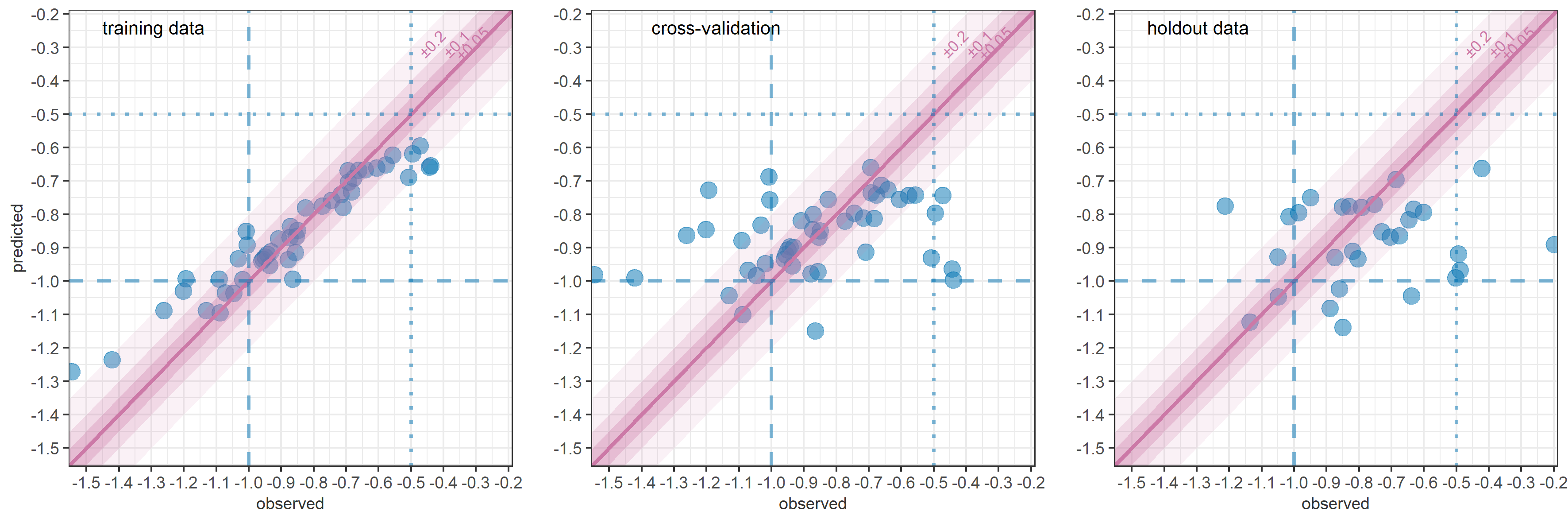


Figure S12. RF-predicted versus observed mean WFH, Somalia, by district-month, on training data, LOOCV and holdout data. Shaded channels indicate different absolute deviance of predictions. Vertical dotted lines denote potentially useful thresholds.

## Predictive accuracy of additional South Sudan models


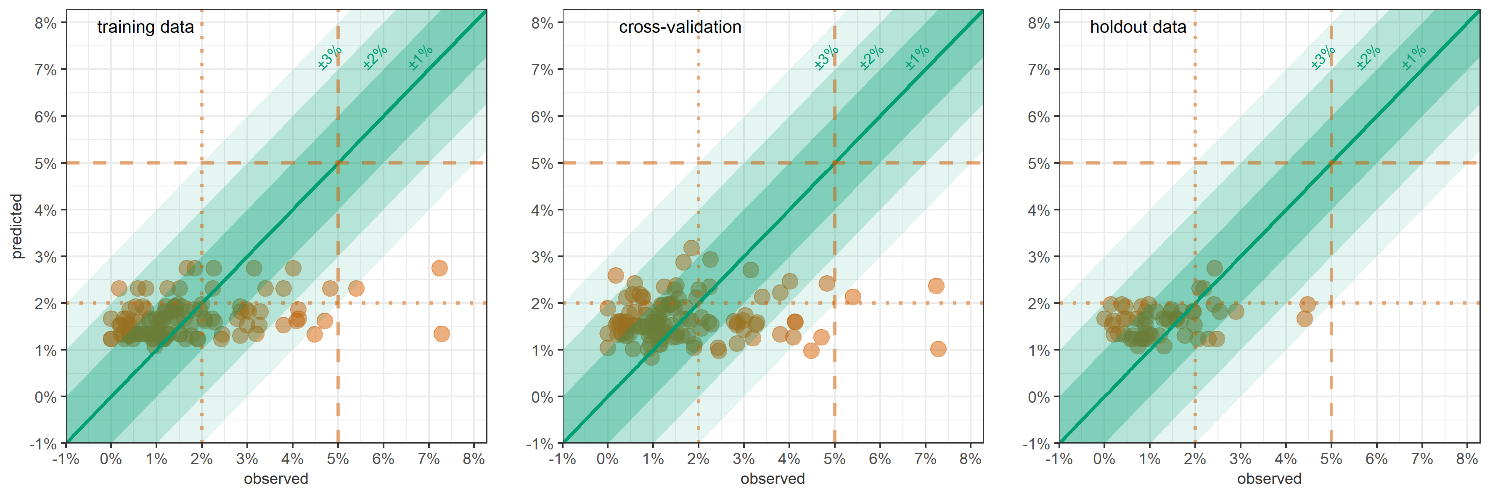


Figure S13. GLM-predicted versus observed SAM (MUAC + oedema) prevalence, South Sudan, by district-month, on training data, LOOCV and holdout data. Shaded channels indicate different absolute deviance of predictions. Vertical dotted lines denote commonly used SAM prevalence thresholds.


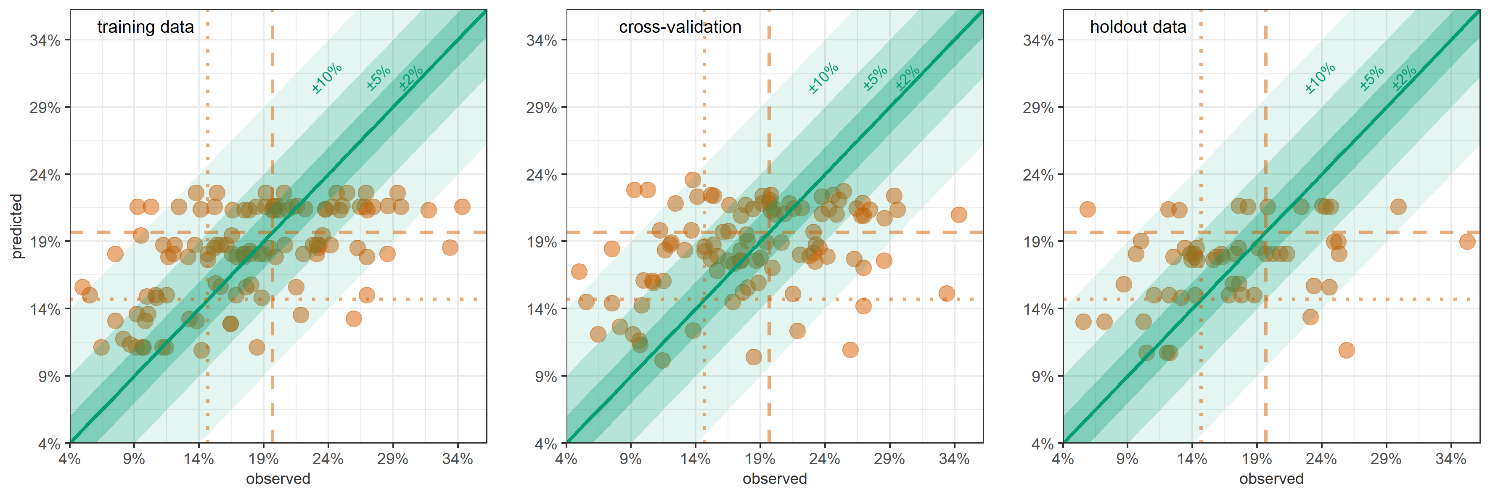


Figure S14. GLM-predicted versus observed GAM (WFH + oedema) prevalence, South Sudan, by district-month, on training data, LOOCV and holdout data. Shaded channels indicate different absolute deviance of predictions. Vertical dotted lines denote commonly used GAM prevalence thresholds.


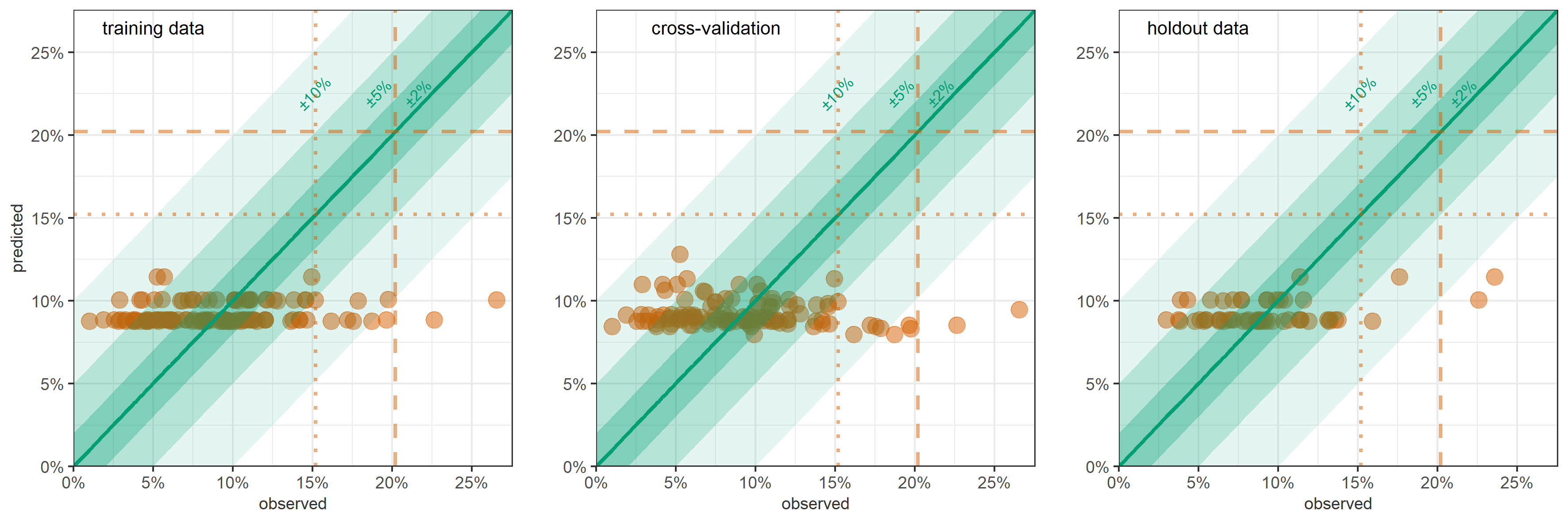


Figure S15. GLM-predicted versus observed GAM (MUAC + oedema) prevalence, South Sudan, by district-month, on training data, LOOCV and holdout data. Shaded channels indicate different absolute deviance of predictions. Vertical dotted lines denote commonly used GAM prevalence thresholds.


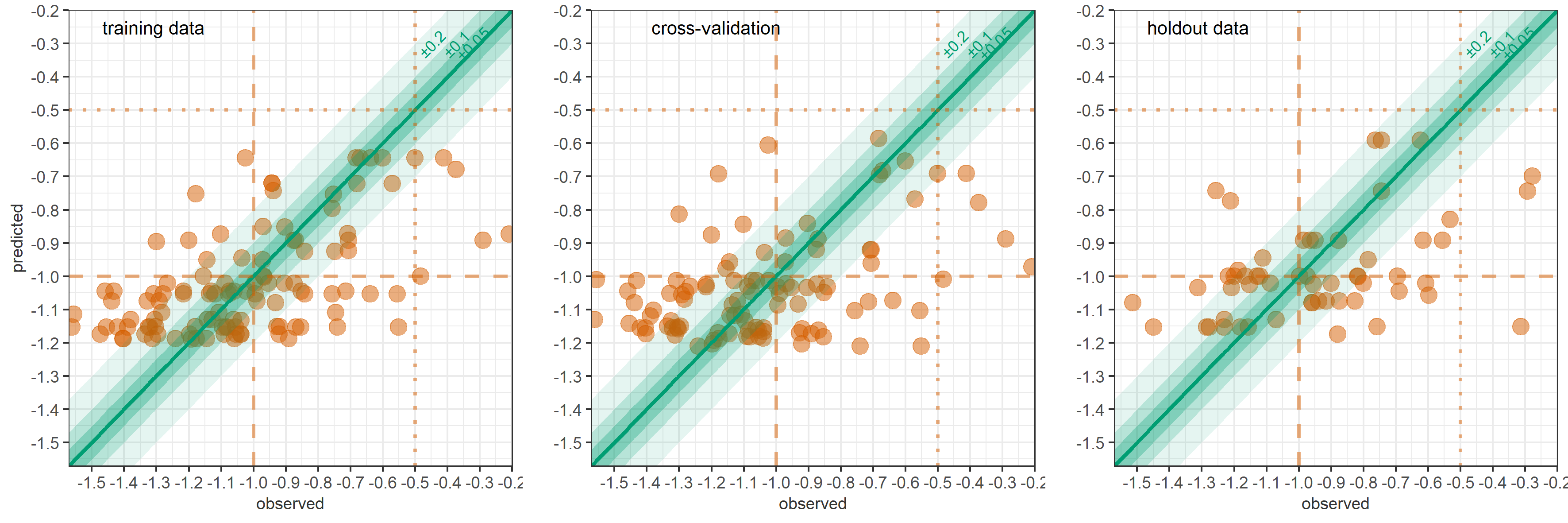


Figure S16. GLM-predicted versus observed mean WFH, South Sudan, by district-month, on training data, LOOCV and holdout data. Shaded channels indicate different absolute deviance of predictions. Vertical dotted lines denote potentially useful thresholds.


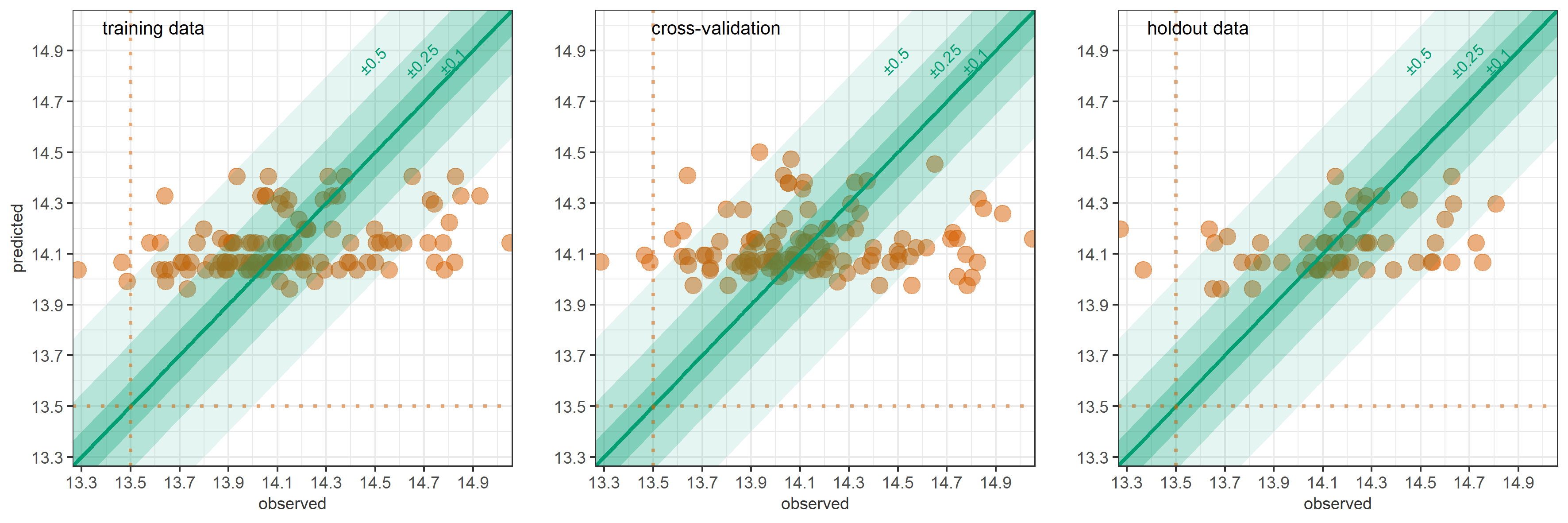


Figure S17. GLM-predicted versus observed mean MUAC, South Sudan, by district-month, on training data, LOOCV and holdout data. Shaded channels indicate different absolute deviance of predictions. Vertical dotted lines denote potentially useful thresholds.

Table S8. Performance of random forest models in South Sudan, by acute malnutrition outcome.

| Statistic | | Binary:  GAM (WFH + oedema) | | Continuous:  WFH | |
| --- | --- | --- | --- | --- | --- |
| Estimation performance | | | | | |
| Mean square error | training data | 0.00069 | | 0.01276 | |
|  | LOOCV | 0.00393 | | 0.07243 | |
|  | holdout data | 0.00331 | | 0.07126 | |
| Relative bias | LOOCV | +14.0% | | +9.2% | |
|  | holdout data | +19.9% | | +21.2% | |
| Relative precision of 95%CI | LOOCV | ±17.2% | | ±14.5% | |
|  | holdout data | ±11.7% | | ±8.5% | |
| Coverage of 95%CI | LOOCV | 39.1% | | 37.4% | |
|  | holdout data | 53.6% | | 46.4% | |
| Coverage of 80%CI | LOOCV | 30.4% | | 27.0% | |
|  | holdout data | 37.5% | | 32.1% | |
| Classification performance by GAM prevalence threshold (n = denominator of percentage) | | | | | |
| Sensitivity  (lower threshold) | LOOCV | ≥15% | 94.9% (79) | n/a | |
|  | holdout data |  | 100.0% (32) |  |  |
| Sensitivity  (upper threshold) | LOOCV | ≥20% | 31.1% (45) |  |  |
|  | holdout data |  | 35.3% (17) |  |  |
| Specificity  (lower threshold) | LOOCV | <15% | 19.4% (36) |  |  |
|  | holdout data |  | 12.5% (24) |  |  |
| Specificity  (upper threshold) | LOOCV | <20% | 80.0% (70) |  |  |
|  | holdout data |  | 87.2% (39) |  |  |
| Top ten predictors by importance | lag | Importance | p-value | Importance | p-value |
| Main livelihood type | n/a | 0.0002 | 0.010 | 0.0083 | 0.010 |
| Terms of trade | 4-6mths prior | 0.0001 | 0.158 | 0.0041 | 0.079 |
|  | 3-5mths prior | 0.0001 | 0.347 | 0.0046 | 0.069 |
|  | 2-4mths prior | not among top ten predictors | | 0.0034 | 0.109 |
| Number of measles vaccine doses administered per population | 3-5mths prior | 0.0001 | 0.337 | 0.0031 | 0.248 |
|  | 2-4mths prior | 0.0003 | 0.050 | 0.0043 | 0.188 |
|  | 1-3mths prior | 0.0002 | 0.238 | 0.0025 | 0.475 |
|  | previous 3mths | 0.0002 | 0.158 | 0.0036 | 0.178 |
| Total rainfall | previous 6mths | 0.0004 | 0.020 | 0.0073 | 0.010 |
| Rate of insecurity events | 4-6mths prior | 0.0001 | 0.099 | not among top ten predictors | |
| Proportion of the population that is internally displaced | n/a | <0.0001 | 0.248 | 0.0023 | 0.139 |


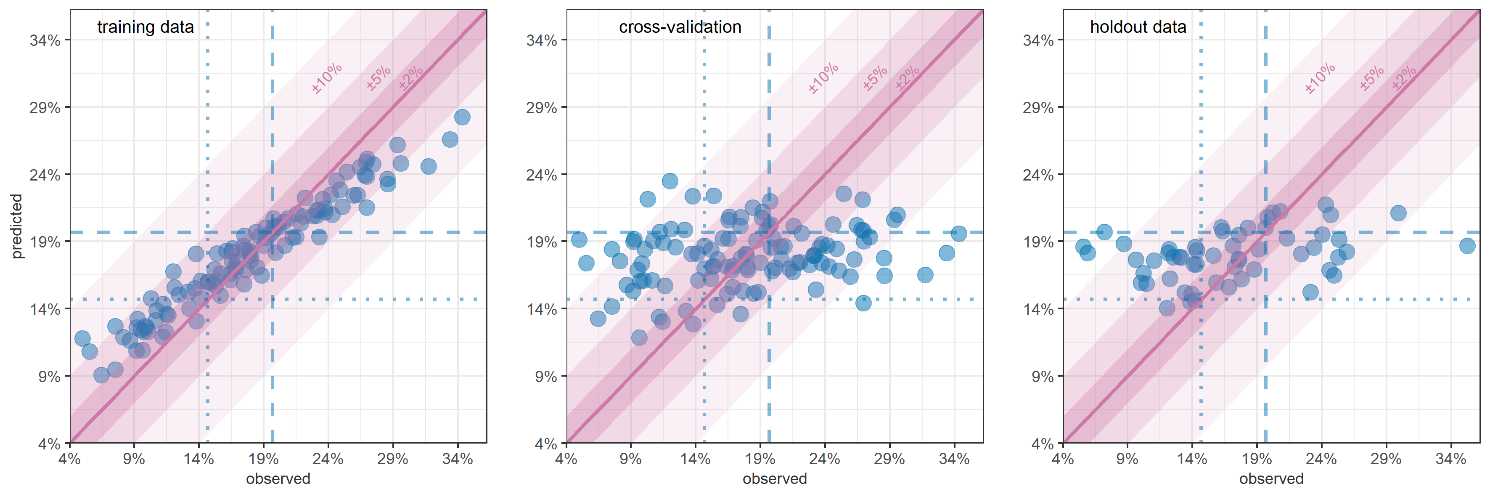


Figure S18. RF-predicted versus observed GAM (WFH + oedema) prevalence, South Sudan, by district-month, on training data, LOOCV and holdout data. Shaded channels indicate different absolute deviance of predictions. Vertical dotted lines denote commonly used GAM prevalence thresholds.


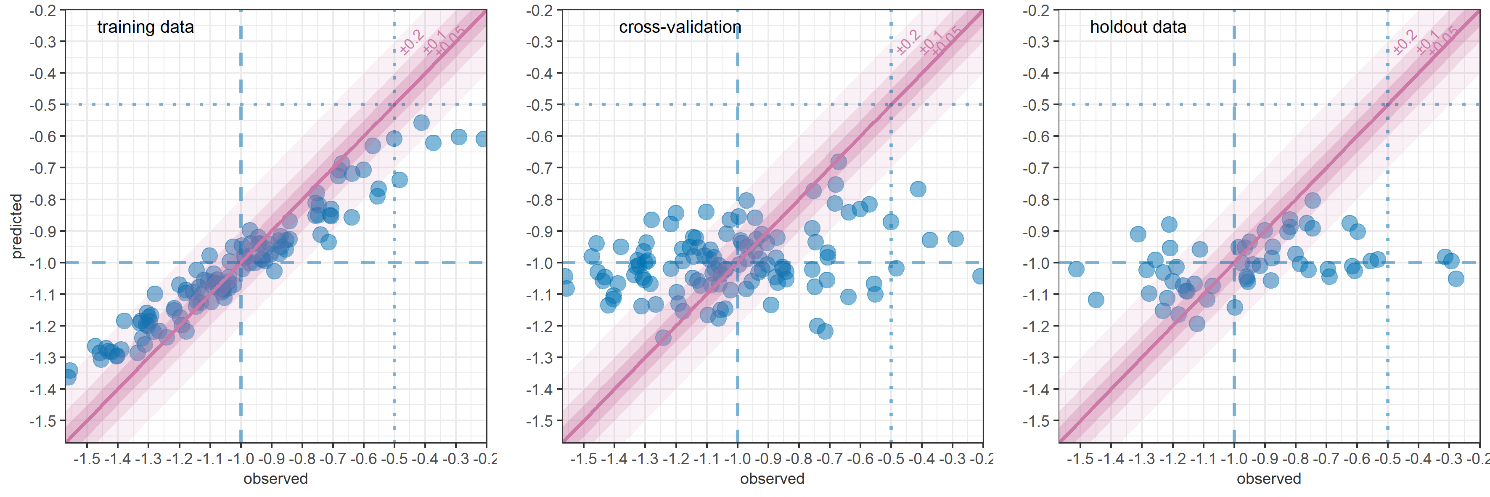


Figure S19. RF-predicted versus observed mean WFH, South Sudan, by district-month, on training data, LOOCV and holdout data. Shaded channels indicate different absolute deviance of predictions. Vertical dotted lines denote potentially useful thresholds.
